# The Rates and the Determinants of Hypertension According to the 2017 Definition of Hypertension by ACC/AHA and 2014 Evidence-Based Guidelines Among Population Aged ≥40 Years Old

**DOI:** 10.5334/gh.914

**Published:** 2021-05-04

**Authors:** Wenzhen Li, Dajie Chen, Shuai Liu, Xiaojun Wang, Xiaojie Chen, Jiafeng Chen, Jing Ma, Fujian Song, Hui Li, Shijiao Yan, Xiaoxv Yin, Shiyi Cao, Yanhong Gong, Junan Liu, Wei Yue, Feng Yan, Chuanzhu Lv, Zhihong Wang, Zuxun Lu

**Affiliations:** 1 Department of Social Medicine and Health Management, School of Public Health, Tongji Medical College, Huazhong University of Science and Technology, Wuhan, CN; 2 Health and Family Planning Commission of Wuhan Municipality, Wuhan, CN; 3 Department of Tuberculosis Prevention, Wuhan Tuberculosis Institution, Wuhan, CN; 4 China Urban Construction Design and Research Institute, Beijing, CN; 5 Department of Software Engineering, School of Cyber Science and Engineering, Wuhan University, Wuhan, CN; 6 Department of Population Medicine, Harvard Medical School, Harvard University, Boston, MA, US; 7 Department of Population Health and Primary Care, Norwich Medical School, University of East Anglia, Norwich, GB; 8 School of International Education, Hainan Medical University, Haikou, CN; 9 Neurology Department, Tianjin Huanhu Hospital, Tianjin, CN; 10 Xuanwu Hospital, Capital Medical University, Beijing, CN; 11 Emergency and Trauma College, Hainan Medical University, Haikou, Hainan, CN; 12 Shenzhen NO.2 People’s Hospital, Shenzhen University, Shenzhen, CN

**Keywords:** geographical distribution, hypertension burden, hypertension prevalence, risk factors, 2017 AHA/ACC guidelines

## Abstract

**Background:**

In November 2017, the American College of Cardiology/American Heart Association (ACC/AHA) updated their definition of hypertension from 140/90 mm Hg to 130/80 mm Hg.

**Objectives:**

We sought to assess the situation of hypertension and the impact of applying the new threshold to a geographically and ethnically diverse population.

**Methods:**

We analyzed selected data on 237,142 participants aged ≥40 who had blood pressure taken for the 2014 China National Stroke Screening and Prevention Project. Choropleth maps and logistic regression analyses were performed to estimate the prevalence, geographical distribution and risk factors of hypertension using both 2017 ACC/AHA guidelines and 2014 evidence-based guidelines.

**Results:**

The present cross-sectional study showed the age- and sex-standardized prevalence of hypertension was 37.08% and 58.52%, respectively, according to 2014 evidence-based guidelines and 2017 ACC/AHA guidelines. The distribution of hypertension and risk factors changed little between guidelines, with data showing a high prevalence of hypertension around Bohai Gulf and in south central coastal areas using either definition. The age- and sex-standardized prevalence of newly labeled as hypertensive was 21.44%. Interestingly, the high prevalence region of newly labeled as hypertensive was found in the north China.

**Conclusion:**

The prevalence of hypertension increased significantly on 2017 ACC/AHA guidelines compared to the prevalence when using 2014 evidence-based guidelines, with high prevalence areas of newly labeled as hypertensive now seen mainly in north China. There need to be correspondingly robust efforts to improve health education, health management, and behavioral and lifestyle interventions in the north.

## Introduction

The 2017 American College of Cardiology/American Heart Association (ACC/AHA) hypertension guidelines for the prevention, detection, evaluation, and management of high blood pressure (BP) in adults define hypertension as systolic blood pressure (SBP) ≥130 mmHg and diastolic blood pressure (DBP) ≥80 mmHg, replacing the previous threshold of 140/90 (SBP/DBP) mmHg as defined by 2014 evidence-based guidelines for managing high BP in adults developed by panel members appointed to the Eighth Joint National Committee (JNC 8) [1,2]. The current change in the 2017 ACC/AHA guidelines was mainly based on epidemiological evidence, indicating that a lower threshold of 130/80 mmHg seemed reasonable as a cutoff for hypertension diagnosis and target BP for hypertension treatment [3,4].

The new guidelines were based on the premise that strict BP lowering could maintain vascular health in early life and protect against cardiovascular disease (CVD) and organ damage later. From this perspective, the new guidelines are of great significance [5,6]. They have prompted intense discussion in many countries [7,8,9] on the effects of an increased number of patients requiring treatment for high BP on healthcare costs and whether the change would improve BP control without substantially increasing treatment side effects. The significance of the new threshold on a global level is unknown.

This is particularly true in China, where the move to update the Chinese hypertension guidelines is controversial given China’s multiethnicity, with the largest population worldwide. During the past decades, the prevalence of hypertension has rapidly increased with treatment characterized by inadequate control [10,11,12]. Opponents argue that China’s economic level and overall disease burden differ from those in the United States. However, data are limited on whether that is the case. Understanding the change in prevalence, distribution, and risk factors of hypertension in China’s population using the new guidelines is significant and may provide important clues on the appropriateness of applying the new guidelines globally.

Therefore, we analyzed the prevalence, geographical distribution, and risk factors of hypertension according to the different diagnostic thresholds using data from the China National Stroke Screening and Prevention Project (CNSSPP) in 2014–2015 [13] to provide baseline data and theoretical basis for the rationality of the new threshold.

## Methods

### Participants and study design

As previously described [14 15], CNSSPP focused on middle-aged and elderly population aged ≥40 years. It was conducted in 200 project areas within 30 provinces and municipalities in mainland China from October 2014 to November 2015 as part of a special project for healthcare reform established by the Ministry of Finance and National Health and Family Planning Commission.

A two-stage stratified cluster sampling method was used. First, the size of the screening population and project areas in each province were determined according to the local population size and total number of counties. Then, an urban community and rural village were selected as primary sampling units from each project area. The population to be screened in each screening unit was determined according to the proportion of urban and rural population aged ≥40 years. Primary screening units were selected by local health administrations according to recommendations from staff in local hospitals. The cluster sampling method was used in every primary screening unit, and ≥85% of local residents aged ≥40 years (born before October 31, 1974) were surveyed in the primary screening process.

The entire screening process comprises two stages. In the primary screening, 726,451 participants were included. Community physicians collected information on participants’ sociodemographic characteristics during in-person interviews. In the second stage, participants were invited for further physical examination, including BP and waist circumference measurements, laboratory tests, electrocardiogram, and carotid ultrasound. Among them, 237,142 participants underwent BP examination with full data. Thus, a total of 237,142 were included in our analyses.

### Data Collection

The study protocol was approved by the ethics committee of the Xuanwu Hospital Institutional Review Board, Capital Medical University (Beijing, China), all methods were performed in accordance with the relevant guidelines and regulations. Written informed consent was obtained from all participants. Screening was conducted by trained clinics in participants’ residential area according to a standard protocol.

SBP and DBP were measured three times consecutively on the upper arm by community physicians using a digital sphygmomanometer (Omron HEM-7201, Omron Company, Kyoto, Japan) in the sitting position after resting for ≥5 min, without speech or movement allowed at measurement. The mean of the three recorded values of SBP and DBP was used in the analyses. For participants whose BP was measured on both arms, the average recorded values were reported separately. After measurement, participants were asked if they had used a prescribed antihypertensive drug in the past two weeks. Community physicians also collected information on medical history and obtained physical measurements.

### Definition of Analysis Variables

Hypertension in 2014 evidence-based guidelines: consistent with JNC 8, hypertension was defined as SBP ≥140 mmHg or DBP ≥90 mmHg or self-reported use of an antihypertensive drug in the past two weeks.

Hypertension in 2017 ACC/AHA guidelines: participants were considered hypertensive if they had a measured SBP ≥130 mmHg or a measured DBP ≥80 mmHg or self-reported use of an antihypertensive drug in the past two weeks.

Newly labeled as hypertensive: participants were nonhypertensive based on the JNC 8 criteria but would be classified as having hypertension in the 2017 ACC/AHA guidelines.

Lack of physical activity was defined as fewer than four sessions of regular physical exercise per week and <30 min/session. BMI was defined as the weight in kilograms divided by the square of the height in meters (kg/m^2^). Family history of stroke was defined as having ≥1 first-degree relative with stroke.

### Statistical Analysis

Statistical analysis was performed in March 2018, and 237,142 participants were included. No missing values for the nine key variables: sex, age, ethnic origin, geographical region (north, northeast, east, south central, southwest, and northwest) [16], residence (urban, rural), lack of physical activity, current smoking, BMI ≥26 kg/m^2^ [15], and family history of stroke. All these variables were included in the binary regression model and were adjusted with other factors.

Age- and sex-standardized prevalence of hypertension was calculated using the distribution of population aged ≥40 years in the 2010 population census of China to describe the distribution of hypertensive population in different subgroups [17]. We assigned 30 provinces and municipalities into corresponding prevalence terciles, first tercile (low-prevalence region), second tercile (moderate-prevalence region), and third tercile (high-prevalence region), after ranking all provinces and municipalities by age- and sex-standardized prevalence. Choropleth maps were drawn to visualize geographic distributions of the prevalence of hypertension. Logistic regression analyses with odds ratios (ORs) and their corresponding 95% confidence intervals (CIs) were performed to systematically estimate the association between hypertension and nine key sociodemographic characteristics (gender, age, ethnic origin, geographical region, urbanity, physical activity, smoke status, BMI, and family history of stroke). Chi-square (χ*^2^*) tests were used to analyze the distribution of key sociodemographic characteristics between different regions of newly labeled as hypertensive. Statistical analyses were conducted using IBM SPSS version 19 for Windows. All *P*-values are two tailed. *P*-values < 0.05 were considered statistically significant.

## Results

Of 237,142 participants included, 45.38% were men. The mean age was 58.96 ± 11.10 (range 40–108) years. Of these, 113,090 met the JNC 8 criteria for hypertension, and 182,412 were classified as hypertensive according to the 2017 ACC/AHA criteria. The age- and sex-standardized prevalence rates of hypertension in the JNC 8 criteria and 2017 ACC/AHA guidelines were 37.08% and 58.52% in the total population, 37.86% and 60.85% in men, and 36.28% and 56.14% in women, respectively. An additional 69,322 people were classified as hypertensive based on the 2017 ACC/AHA criteria, and the age- and sex-standardized prevalence rate of newly labeled as hypertensive was 21.44% in the total population, 22.99% in men, and 19.86% in women (Table 1). Regardless of the guideline applied, the age- and sex-standardized prevalence rate of hypertension increased significantly with age (*P* < 0.001 in both comparisons), and male participants and residents of the northeast, east, or south-central regions and urban inhabitants had significantly higher prevalence of hypertension (*P* < 0.001); however, the age- and sex-standardized prevalence rate of newly labeled as hypertensive in 2017 ACC/AHA guidelines significantly decreased with age (*P* < 0.001), and the prevalence of newly labeled as hypertensive was significantly higher in people living in the north or northwest regions than in those living in the northeast, east, south-central, or southwest regions (*P* < 0.001).

**Table 1:** Standardized prevalence of hypertension by selected demographic characteristics.

Characteristic	No. of Participants	2014 Evidence-Based Guidelines	2017 ACC/AHA Guidelines	Newly Labeled as Hypertensive

All population	237,142	113,090(37.08)	182,412 (58.52)	69,322 (21.44)
Gender				
Male	107,625 (45.38)	51,790 (37.86)	85,165 (60.85)	33,375 (22.99)
Female	129,517 (54.62)	61,300 (36.28)	97,247 (56.14)	35,947 (19.86)
Age (years)				
40–44	23,754 (10.01)	5,211 (19.98)	14,269 (48.47)	9,058 (28.49)
45–49	32,798 (13.83)	10,331 (28.59)	22,137 (54.35)	11,806 (25.76)
50–54	35,375 (14.91)	14,603 (37.23)	26,209 (59.57)	11,606 (22.34)
55–59	32,955 (13.89)	15,857 (43.38)	25,737 (62.65)	9,880 (19.27)
60–64	38,344 (16.16)	21,144 (49.59)	31,435 (65.68)	10,291 (16.09)
65–69	30,359 (12.80)	18,513 (54.84)	25,614 (67.51)	7,101 (12.67)
70–74	20,513 (8.65)	13,165 (57.71)	17,650 (68.84)	4,485 (11.13)
75–79	13,422 (5.65)	8,469 (56.76)	11,331 (67.53)	2,862 (10.77)
≥80	9,622 (4.05)	5,797 (54.24)	8,030 (66.75)	2,233 (12.51)
Ethnic origin*				
Han	229,997 (96.98)	109,713 (37.03)	176,944 (58.49)	67,231 (21.46)
Minority	7,145 (3.02)	3,377 (38.75)	5,468 (59.29)	2,091 (20.54)
Geographical region				
North	55,981 (23.61)	23,921 (32.71)	42,468 (60.41)	18,547 (27.70)
Northeast	20,363 (8.59)	10,634 (41.64)	16,645 (60.99)	6,011 (19.35)
East	60,707 (25.60)	33,712 (42.70)	49,621 (62.02)	15,909 (19.32)
South central	39,936 (16.84)	21,648 (44.15)	31,483 (61.69)	9,835 (17.54)
Southwest	30,578 (12.89)	11,659 (27.82)	20,269 (50.09)	8,610 (22.27)
Northwest	29,577 (12.47)	11,516 (31.27)	21,926 (58.94)	10,410 (27.67)
Urbanity				
Urban	122,943 (51.84)	54,187 (40.45)	90,229 (61.76)	36,042 (21.31)
Rural	114,199 (48.15)	58,903 (33.98)	92,183 (55.52)	33,280 (21.54)

*Notes*: Estimated rates of the prevalence of hypertension were adjusted by level of stroke risk in all CNSSPP participants. and weighted by 2010 population in China.

**Figure 1:**
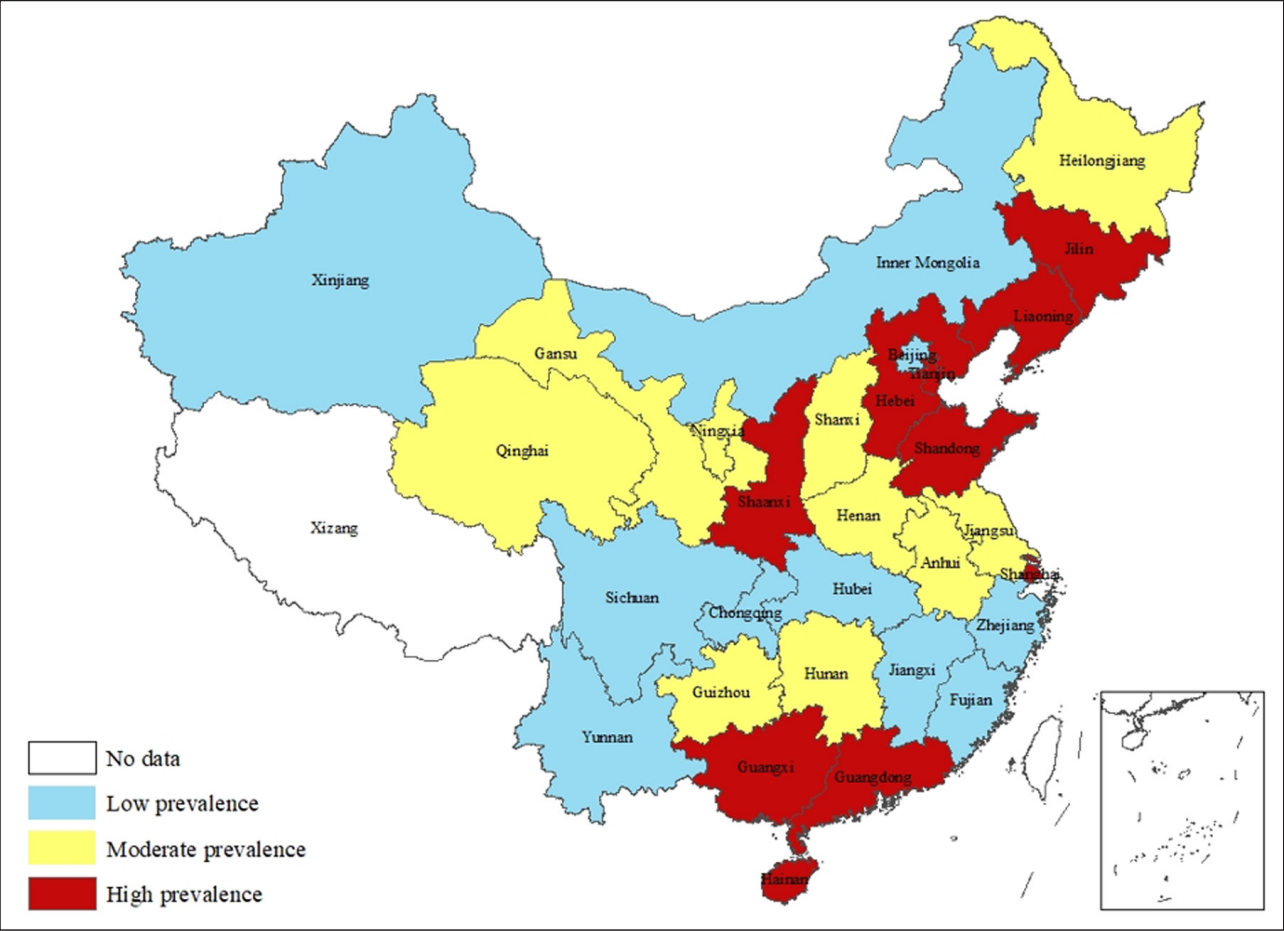
Prevalence of Hypertension in 2017 ACC/AHA Guidelines.

**Figure 2:**
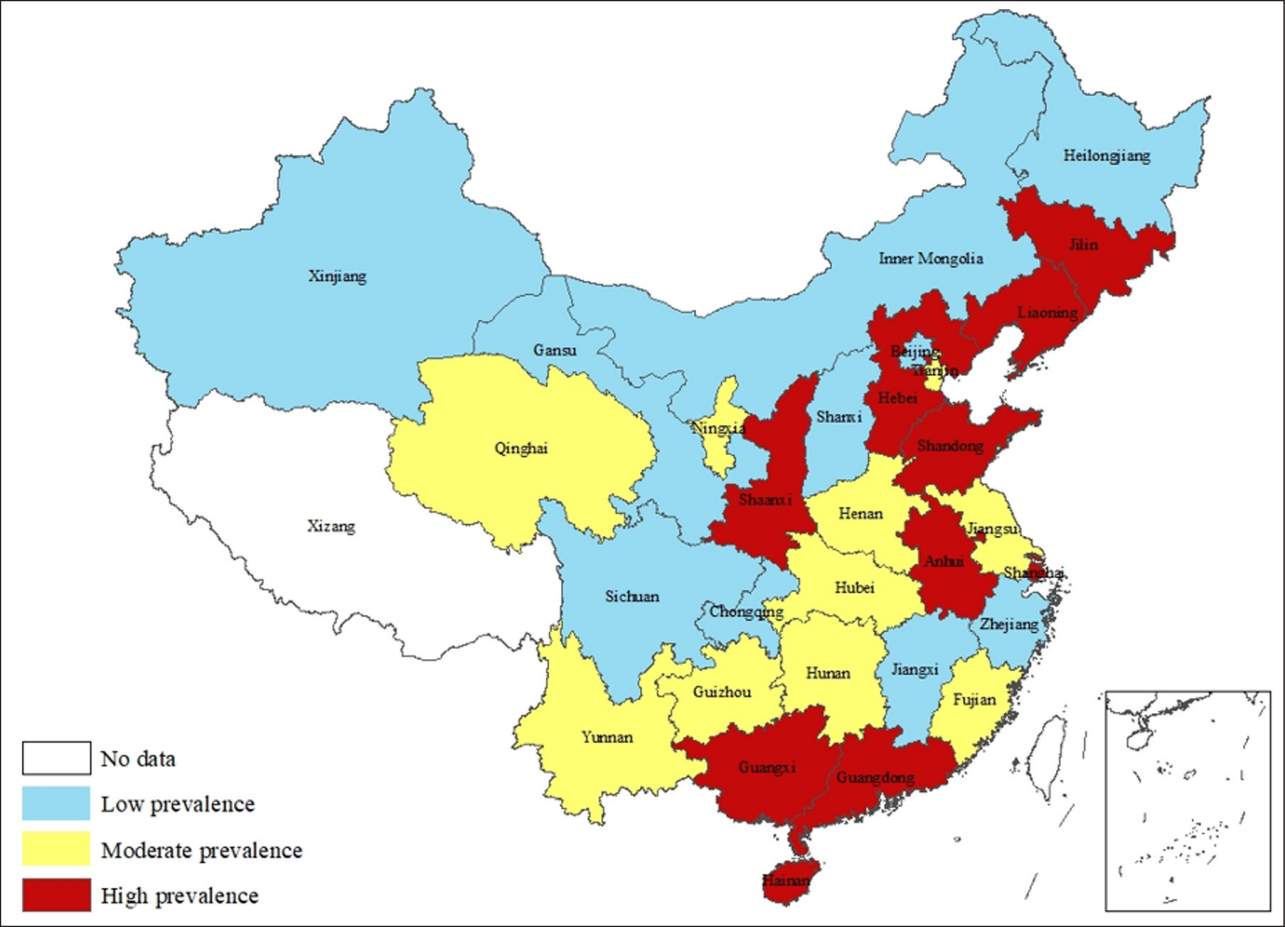
Prevalence of Hypertension in 2014 Evidence-Based Guidelines.

**Figure 3:**
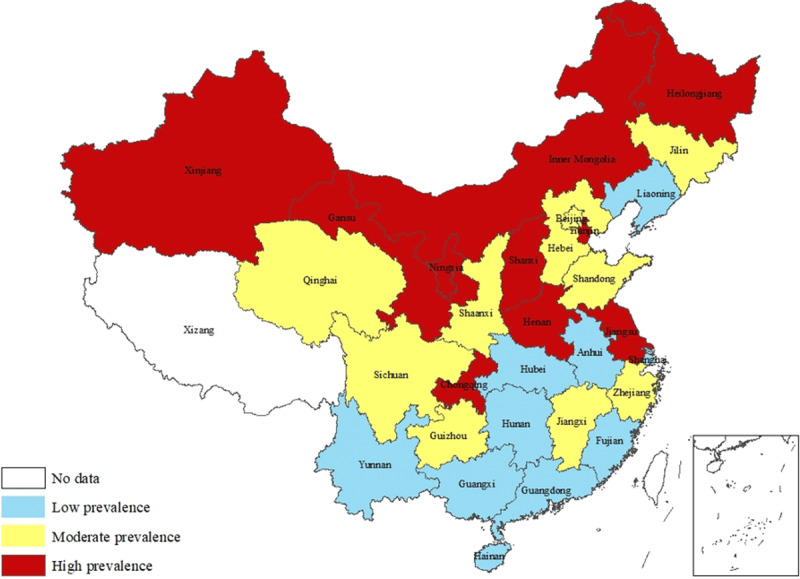
Prevalence of Newly Labeled as Hypertensive.

Geographic distributions of prevalence of hypertension are shown in Figures 1, 2, 3. High-prevalence regions of hypertension in the 2017 ACC/AHA guidelines were concentrated around Bohai Gulf and in south-central coastal areas (Figure 1), almost unchanged compared with their distribution under the 2014 evidence-based guidelines (Figure 2). North-south gradient of prevalence of newly labeled as hypertensive is shown in Figure 3, with higher prevalence regions of newly labeled as hypertensive clustered in the north.

Logistic regression models identified several characteristics associated with hypertension (Table 2). Females have a lower odds ratio (OR) of hypertension (0.96, 0.93–0.98 for 2014 evidence-based guidelines; 0.80, 0.78–0.83 for 2017 ACC/AHA guidelines; 0.74, 0.71–0.77 for newly labeled as hypertensive), and older people (1.50 to 5.54 for 2014 evidence-based guidelines; 1.27 to 3.33 for 2017 ACC/AHA guidelines; 1.13 to 1.61 for newly labeled as hypertensive), urban inhabitants (1.29, 1.27–1.31 for 2014 evidence-based guidelines; 1.42, 1.39–1.44 for 2017 ACC/AHA guidelines; 1.34, 1.32–1.36 for newly labeled as hypertensive), those living in the east (1.79, 1.76–1.81 for 2014 evidence-based guidelines; 1.42, 1.39–1.45 for 2017 ACC/AHA guidelines; 1.07, 1.03–1.10 for newly labeled as hypertensive) or south-central (1.85, 1.82–1.88 for 2014 evidence-based guidelines; 1.22, 1.18–1.25 for 2017 ACC/AHA guidelines) region, and those with stroke risk factors (lack of physical activity, current smoker, BMI ≥26 kg/m^2^, family history of stroke) have a higher OR of hypertension. Ethnic minority was significantly associated with an increased OR of hypertension in 2014 evidence-based guidelines (0.82, 0.76–0.87); however, the association was not found in 2017 ACC/AHA guidelines (1.01, 0.95–1.07). Most risk factors of newly labeled as hypertensive were consistent with those of hypertension. The OR of newly labeled as hypertensive was higher in northern regions than in southern regions (0.81, 0.77–0.84). We further analyzed the distribution of nine key variables to explain the difference in geographic distributions of newly labeled as hypertensive (Table 3). Generally, the distribution of the nine key variables in low-, moderate-, and high-prevalence regions was significantly different. The proportion of people who were female, elderly, ethnic Han, and rural inhabitants and had stroke risk factors in high-prevalence regions was lower than those in low- and moderate-prevalence regions (*P* < 0.001).

**Table 2:** Risk factors associated with hypertension.

Characteristic	2014 Evidence Based Guidelines	2017 ACC/AHA Guidelines	Newly Labeled as Hypertensive

Gender (reference: male)			
Female	0.96 (0.93, 0.98)	0.80 (0.78, 0.83)	0.74 (0.71, 0.77)
Age (reference: 40–44 years)			
45–49	1.50 (1.45, 1.54)	1.27 (1.23, 1.31)	1.13 (1.09, 1.17)
50–54	2.16 (2.12, 2.19)	1.66 (1.62, 1.69)	1.23 (1.19, 1.27)
55–59	2.74 (2.70, 2.79)	1.98 (1.94, 2.02)	1.39 (1.35, 1.43)
60–64	3.45 (3.41, 3.49)	2.40 (2.36, 2.44)	1.50 (1.46, 1.55)
65–69	4.35 (4.31, 4.39)	2.79 (2.74, 2.83)	1.51 (1.47, 1.56)
70–74	5.31 (5.26, 5.35)	3.33 (3.28, 3.38)	1.61 (1.56, 1.67)
75–79	5.54 (5.49, 5.59)	3.12 (3.06, 3.17)	1.43 (1.37, 1.50)
≥80	5.26 (5.21, 5.32)	3.12 (3.05, 3.18)	1.51 (1.44, 1.59)
Ethnic origin (reference: minority)			
Han	0.82 (0.76, 0.87)	1.01 (0.95, 1.07)*	1.14 (1.07, 1.21)
Geographical region (reference: north)			
Northeast	1.09 (1.06, 1.13)	1.11 (1.06, 1.15)	1.08 (1.03, 1.13)
East	1.79 (1.76, 1.81)	1.42 (1.39, 1.45)	1.07 (1.03, 1.10)
South central	1.85 (1.82, 1.88)	1.22 (1.18, 1.25)	0.81 (0.77, 0.84)
Southwest	0.93 (0.90, 0.96)	0.68 (0.65, 0.72)	0.63 (0.59, 0.67)
Northwest	1.05 (1.02, 1.08)	1.02 (0.99, 1.06)*	0.99 (0.95, 1.03)*
Urbanity (reference: rural)			
Urban	1.29 (1.27, 1.31)	1.42 (1.39, 1.44)	1.34 (1.32, 1.36)
Stroke risk factors			
Lack of physical activity (reference: no)	1.27 (1.25, 1.29)	1.15 (1.13, 1.17)	1.00 (0.98, 1.03)*
Current smoker (reference: no)	1.45 (1.42, 1.48)	1.22 (1.19, 1.25)	1.04 (1.01, 1.09)
BMI ≥ 26 kg/m^2^ (reference: no)	2.46 (2.44, 2.48)	2.31 (2.28, 2.33)	1.53 (1.50, 1.56)
Family history of stroke (reference: no)	1.86 (1.84, 1.89)	1.67 (1.63, 1.70)	1.20 (1.16, 1.24)

**BMI:** body-mass index. * represents *P* value > 0.05, there is no statistical difference compared to reference group.

**Table 3:** Distribution of selected demographic characteristics between different prevalence regions of newly labeled as hypertensive.

Characteristic		Low Prevalence Region	Moderate Prevalence Region	High Prevalence Region	*P* Value

Gender	Male	18950 (45.10)	37326 (44.58)	51349 (46.09)	0.001
	Female	23064 (54.90)	46402 (55.42)	60051 (53.91)	
Age (years)	40–44	3098 (7.37)	8260 (9.87)	12396 (11.13)	0.001*
	45–49	4792 (11.41)	11213 (13.39)	16793 (15.07)	
	50–54	6084 (14.48)	11930 (14.25)	17361 (15.58)	
	55–59	5246 (12.49)	11650 (13.91)	16059 (14.42)	
	60–64	7371 (17.54)	13935 (16.64)	17038 (15.29)	
	65–69	6270 (14.92)	11393 (13.61)	12696 (11.40)	
	70–74	4233 (10.08)	7687 (9.18)	8593 (7.71)	
	75–79	2836 (6.75)	4549 (5.43)	6037 (5.42)	
	≥80	2084 (4.96)	3111 (3.72)	4427 (3.97)	
Ethnic origin	Minority	725 (1.73)	2547 (3.04)	3873 (3.48)	0.001*
	Han	41289 (98.27)	81181 (96.96)	107527 (96.52)	
Urbanity	Urban	21704 (51.66)	40553 (48.43)	60686 (54.48)	0.001*
	Rural	20310 (48.34)	43175 (51.57)	50714 (45.52)	
Lack of physical activity	No	28002 (66.65)	59547 (71.12)	84027 (75.43)	0.001*
	Yes	14012 (33.35)	24181 (28.88)	27373 (24.57)	
Current smoker	No	31989 (76.14)	66190 (79.05)	93752 (84.16)	0.001*
	Yes	10025 (23.86)	17538 (20.95)	17648 (15.84)	
BMI ≥ 26 kg/m^2^	No	29288 (69.71)	53786 (64.24)	80891 (72.61)	0.001*
	Yes	12726 (30.29)	29942 (35.76)	30509 (27.39)	
Family history of stroke	No	34108 (81.18)	69905 (83.49)	99455 (89.28)	0.001*
	Yes	7906 (18.82)	13823 (16.51)	11945 (10.72)	

**BMI:** body-mass index. χ^2^ tests were used to analysis distribution of selected demographic characteristics between different prevalence regions of newly labeled as hypertensive. *P* value > 0.05, there is no statistical difference. * represents a statistical difference between any two regions.

## Discussion

This study revealed that hypertension was a significant health problem in the Chinese population aged ≥40 years old. According to the 2014 evidence-based guidelines, the age- and sex-standardized prevalence rate of hypertension was 37.08%. The results were similar to hypertension prevalence in the China Health and Retirement Longitudinal Study (CHARLS), Prospective Urban Rural Epidemiology study, and China Patient-Centered Evaluative Assessment of Cardiac Events (PEACE) Million Persons Project [10,18,19] (41.7%, 41.9%, and 37.2%, respectively), indicating that the screening level and results in the CNSSPP were reliable.

Based on the 2017 ACC/AHA guidelines, prevalence of hypertension increased to 58.52%. Relevant studies on the prevalence of hypertension in the middle-aged and elderly population based on 2017 ACC/AHA hypertension guidelines were scarce. CHARLS demonstrated that, with adoption of the 2017 ACC/AHA hypertension guidelines, the prevalence of hypertension would increase to 55% in the population aged 45–75 years [20]. A recent China Hypertension Survey (CHS) with 451,755 residents aged ≥18 years in 31 provinces showed that the prevalence of hypertension based on the 2017 ACC/AHA guidelines was twice as high as that based on JNC 8 guidelines [21]. In line with the CHARLS and CHS, the prevalence of hypertension in middle-aged and older adults also sharply increased based on 2017 ACC/AHA guidelines in the present study, almost 1.6 times higher than that in JNC 8 guidelines.

### Geographical Distribution of Hypertension and Risk Factors

This study showed that high-prevalence population was mainly distributed in the northeast, east, and south-central regions, especially around Bohai Gulf and in southern coastal cities under both 2014 JNC 8 and 2017 ACC/AHA guidelines. The results of the China PEACE Million Persons Project showed that the prevalence of hypertension in the east, central, and west regions decreased, and the difference between the eastern and central regions was marginally relative [10]. Another survey of the China Chronic Disease and Risk Factors Surveillance in 2014 demonstrated that the hypertensive population was mostly concentrated in the areas around Bohai Gulf [7]. Our results were consistent with those studies and the geographical distribution characteristics of high BP in adolescents [22]. The economic development is positively correlated with the prevalence of hypertension [16,23]. Economies in the eastern and central regions are more developed than that in western regions, possibly explaining to some extent the geographical difference in hypertension in the present study. Moreover, low temperature in north regions difference in health care access and eating habits such as relatively high salt intake in the Bohai Gulf region might also contribute to geological difference in hypertension prevalence [24]. Considering that eastern and south coastal regions provide the best access to treatment in China, behavior and lifestyle intervention might need to be prioritized in these provinces.

Consistent with other studies [25,26,27], the present study showed that lack of physical activity, smoking, overweight, and obesity are constant risk factors for hypertension. Regardless of the hypertension guidelines followed, these identified risk factors should be always emphasized in high-risk populations with interventions aimed at increasing health education and changing behavior and lifestyle to reduce the risk of developing hypertension.

### Perspective for Appropriateness of the 2017 ACC/AHA Guidelines

Our study provided important theoretical basis for appropriateness of the new definition. When we applied the 130/80 mmHg threshold in this study, the prevalence of hypertension increased from 37.08% to 58.52%. Targeting 130/80 mmHg will increase treatment intensity, potentially increasing the cost for medication, burden on the healthcare system, and potential drug-related adverse reactions. One study suggests that compared with standard hypertension control (140/90 mmHg), intensive BP control (133/76 mmHg) in Chinese adults aged 35 ~ 84 years with hypertension would lead to an increase of 136.9 billion Chinese Yuan in treatment costs and 17% more hypotensive events in the next 10 years [28]. Currently, if we apply 2017 ACC/AHA guidelines, the increased direct cost due to the increased treatment intensity and prevalence would bring a major challenge in the Chinese healthcare system. However, in the long run, the totality is cost-saving and lifesaving [9].

High-prevalence regions of newly labeled as hypertensive were mainly distributed in China’s northern provinces. We also found a clear north-south gradient. This is in line with a study showing that China’s Stroke Belt also runs in the north [29]. Hypertension is the leading cause of stroke, and approximately 80% of stroke events are related to hypertension. Because antihypertensive treatment potentially reduces stroke events in patients with hypertension by 35%–45% [2,30], early identification of individuals newly labeled as hypertensives in the Stroke Belt and application of the 130/80 mmHg threshold may decrease the number of strokes by raising awareness on prevention.

We compared the distribution of demographic characteristics of high-, moderate-, and low-prevalence regions to determine the reasons for geographical distribution differences of newly labeled as hypertensive and found factors, including sex, age, and urbanity, could partly explain the north-south gradient. Furthermore, we speculated ambient temperature and ethnic origin might play important roles in this phenomenon because the development of hypertension is regulated by both environmental and genetic factors [31]. Generally, the ambient temperature is lower in northern regions than in other regions, and lower temperature may increase adult BP [32], possibly partly explaining the clear north-south gradient of newly labeled as hypertensive. We speculated that peripheral vasoconstriction due to low temperature may explain this phenomenon. The annual average temperature of north China was 8.55 [33] and similar to those of North America and northern Europe, of which the annual average temperature was 10.89 and 11.56, respectively [34]. This suggests that the new guidelines are more appropriate in areas with relatively low temperatures. Furthermore, as shown in the present study, the proportion of ethnic minorities, mainly Mongolian, Hui, and Uygur, in the region was higher than in low- and moderate-prevalence regions. Similar genetic background [35] and dietary habits exist between these ethnic minorities and European populations [36]. We speculated that the new standard may be of great significance in North American and European countries.

Therefore, our study provided some important clues to the appropriateness of the new guidelines, and further targeted studies are needed to examine our views. Considering the effect of ethnic factors on hypertension, like the BMI classification criteria, which also take the difference of physique and fat distribution into account in different ethnic populations, the classification criteria of the threshold of hypertension should be different among various ethnic and geographical populations.

### Strengths and Limitations

The present study demonstrated marked variations in the prevalence of hypertension between the 2014 evidence-based guidelines and 2017 ACC/AHA guidelines. Selected contextual factors were found to explain some of these variations. Our findings have important implications for policies aiming to enhance prevention and control of hypertension in China and provide some theoretical basis for the usefulness and appropriateness of the new hypertension guidelines.

The study’s limitations include its restriction to China. Further study of other potential contributing factors and large national surveys among other populations should be conducted to assess the global appropriateness of the new guidelines. Screening data for the Xizang Autonomous Region were missing. The effect on the results may not be greatly significant as the sparsely populated region accounts for only 0.23% of China’s total population. Last but not the least, education level was not collected in the study.

## Conclusion

This study shows that hypertension among middle-aged and elderly residents is highly prevalent in China and efforts to improve the effectiveness of hypertension management are needed. Considering that the change in the prevalence of hypertension between JNC 8 and 2017 ACC/AHA guidelines is robust in China’s northern regions, there need to be correspondingly robust efforts to improve health education, health management, and behavioral and lifestyle interventions in the north.

## Abbreviations

ACC/AHA = American College of Cardiology/American Heart Association

BP = blood pressure

BMI = body-mass index

CNSSPP = China National Stroke Screening and Prevention Project Study

CVD = cardiovascular disease

CHARLS = China Health and Retirement Longitudinal Study

CHS = China Hypertension Survey

DBP = diastolic blood pressure

JNC = Joint National Committee

PEACE = China Patient-Centered Evaluative Assessment of Cardiac Events Million Persons Project

SBP = systolic blood pressure
